# Supplementary material for: 40 Hz Audiovisual Stimulation Improves Sustained Attention and Related Brain Oscillations
Source: bioRxiv. 2025 Aug 25:2025.08.25.671937. Preprint. [Version 1] doi: 10.1101/2025.08.25.671937 (PMC12407815; doi:10.1101/2025.08.25.671937)
Supplement: Supplement 1 [file media-1.pdf]

SUPPLEMENTARY INFORMATION

40 Hz Audiovisual Stimulation Improves Sustained Attention and Related Brain Oscillations

Authors: Matthew K. Attokaren*[1], Lu Zhang*[1,2], Sindhura Mettupalli[1], Annabelle C. Singer[1]

Contents:
3 Tables
4 Figures

TABLES

Table 1. Demographics of Stimulation Subjects per Group

Characteristics	40Hz	Random	Light	40Hz vs Random p-value	40Hz vs Constant p-value
	(n = 21)	(n = 22)	(n = 19)		
Years of Age (mean, (s.d))	20 (1.4)	20 (1.6)	21 (4.0)	0.42	0.29
Female Sex (n (%))	7 (33)	13 (59)	10 (52)	0.13	0.61
Years of Education (mean, (s.d))	14 (1.7)	15 (1.5)	16 (2.3)	0.38	0.12
Race: Caucasian (n (%))	9 (43)	10 (45)	8 (42)	0.99	0.88

Years of Age, Years of Education, and all Percentage values are rounded to the nearest integer. Standard deviation values are rounded to the nearest tenth. Student T-tests were used to compare Years of Age and Years of Education. Chi-square tests were used to compare Female Sex, and Race. P-values were not corrected for multiple comparisons.

Table 2. Difference between 40 Hz & Light Group and 40 Hz & Random Groups

Band	Channel	40 vs Light	40 vs Random
**Delta**	Fp1	0.048	0.048
**Delta**	Cz	0.0805	0.031
**Delta**	Oz	0.3931	0.048
**Theta**	Fp1	0.5278	0.2967
**Theta**	Cz	0.7102	0.8719
**Theta**	Oz	0.5614	0.6218
**Alpha**	Fp1	0.2967	0.2967
**Alpha**	Cz	0.5567	0.0805
**Alpha**	Oz	0.5567	0.5565
**Beta**	Fp1	0.0855	0.1416
**Beta**	Cz	0.4385	0.2863
**Beta**	Oz	0.8719	0.6098
**Slow Gamma**	Fp1	0.2967	0.2863
**Slow Gamma**	Cz	0.3786	0.4164
**Slow Gamma**	Oz	0.6218	0.2967

T-test comparing the power between 40 Hz and Light groups and 40 Hz and Random groups in each frequency band on the channels of interest. P-values are FDR-corrected for 30 comparisons (3 channels, 5 frequency bands, 2 comparisons).

Table 3. Power-Behavior Correlation

		PSD-Acc		PSD-RT	
**Channel**	**Band**	**rho**	**p-value**	**rho**	**p-value**
Fp1	Delta	-0.495	0.0005	0.4895	0.0008
Cz	Delta	-0.4759	0.0005	0.3359	0.0358
Oz	Delta	-0.457	0.0007	0.3228	0.0358
Fp1	Theta	-0.1421	0.3283	0.1943	0.232
Cz	Theta	-0.0765	0.5874	-0.0077	0.9958
Oz	Theta	-0.2226	0.1284	0.1079	0.539
Fp1	Alpha	0.3517	0.0164	-0.3337	0.0358
Cz	Alpha	0.3428	0.0167	-0.2108	0.2119
Oz	Alpha	0.2528	0.084	-0.1815	0.2513
Fp1	Beta	0.2517	0.084	-0.3136	0.0358
Cz	Beta	0.1199	0.3982	0.0277	0.9958
Oz	Beta	0.1518	0.315	-0.0021	0.9958
Fp1	Slow Gamma	0.1607	0.3068	-0.2081	0.2119
Cz	Slow Gamma	0.0457	0.72	0.0007	0.9958
Oz	Slow Gamma	0.2583	0.084	-0.1591	0.313

Spearman's rank correlation rho-values and p-values for correlations between power and accuracy and between power and reaction time. p-values are FDR-corrected for 15 comparisons (3 channels, 5 frequency bands).

SUPPLEMENTARY FIGURES

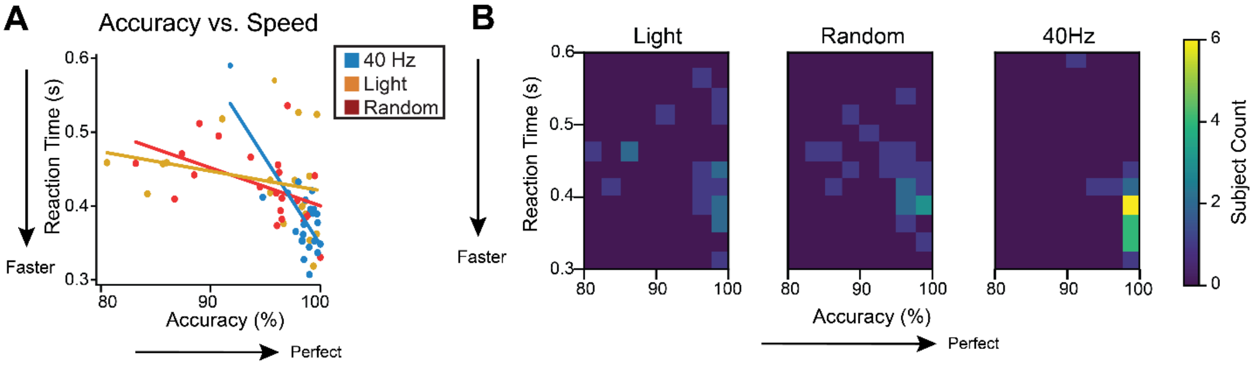

Supplementary Figure 1. Accuracy versus speed relationship during PVT

(A) Average accuracy versus average reaction time per subject for those that underwent 40 Hz flicker (blue; Spearman's rank correlation: Spearman's rho=-0.412, p-value= 0.072, n=21), Random (red; Spearman's rank correlation: Spearman's rho=-0.569, p-value=0.006, n=22), and Constant Light (gold; Spearman's rank correlation: Spearman's rho=-0.412, p-value=0.079, n=19). Each dot is one subject. Lines indicate best linear fit (first-degree polynomial fit). Higher accuracy was not correlated with slower reaction times. Instead, there was a trend of a correlation (40Hz and light groups) or a significant correlation (Random group) in the opposite direction (faster reaction time was correlated with higher accuracy).

(B) Heatmap of average accuracy versus average reaction time per stimulation group with more yellow colors indicating more subjects in that bin.

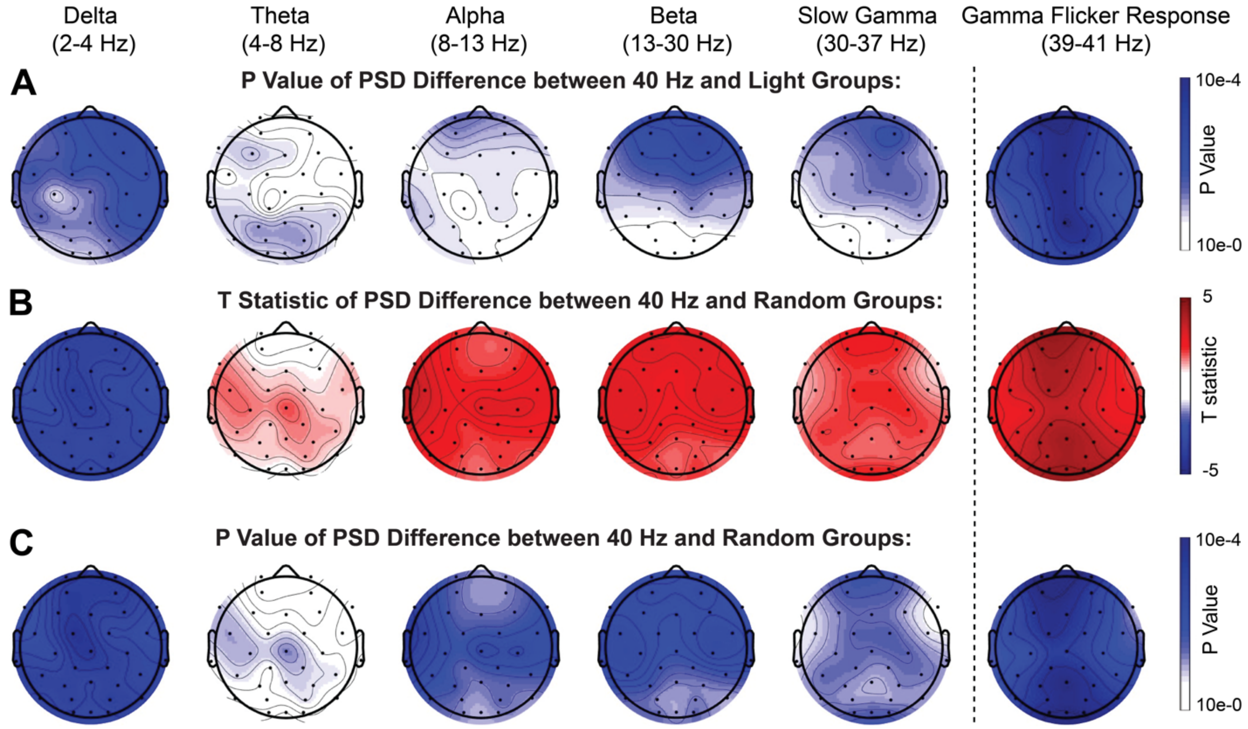

Supplementary Figure 2. 40 Hz flicker decreases delta activity during a vigilance task
(A) maps of p-values (not corrected for multiple comparisons) for PSD differences from comparing the PSD of the 40 Hz group and Light groups during the attention period.
(B) PSD difference map showing the T statistic of the difference between the PSD of the 40 Hz group and Random groups during the attention period.
(C) maps of p-values (not corrected for multiple comparisons) for PSD differences from comparing the PSD of the 40 Hz group and Random groups during the attention period.

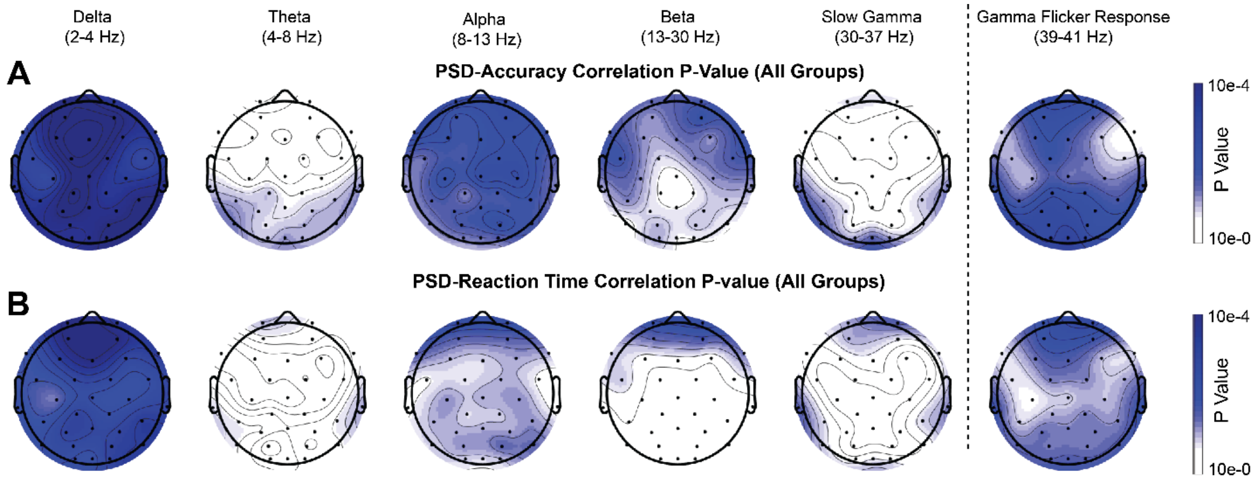

Supplementary Figure 3. Power-behavior correlation maps of p-values.

(A) Maps of p-values (not corrected for multiple comparisons) for power-accuracy correlation across all subjects in all groups. Spearman's rank correlation.

(B) Maps of p-values (not corrected for multiple comparisons) for power-reaction time correlation across all subjects in all groups. Spearman's rank correlation.

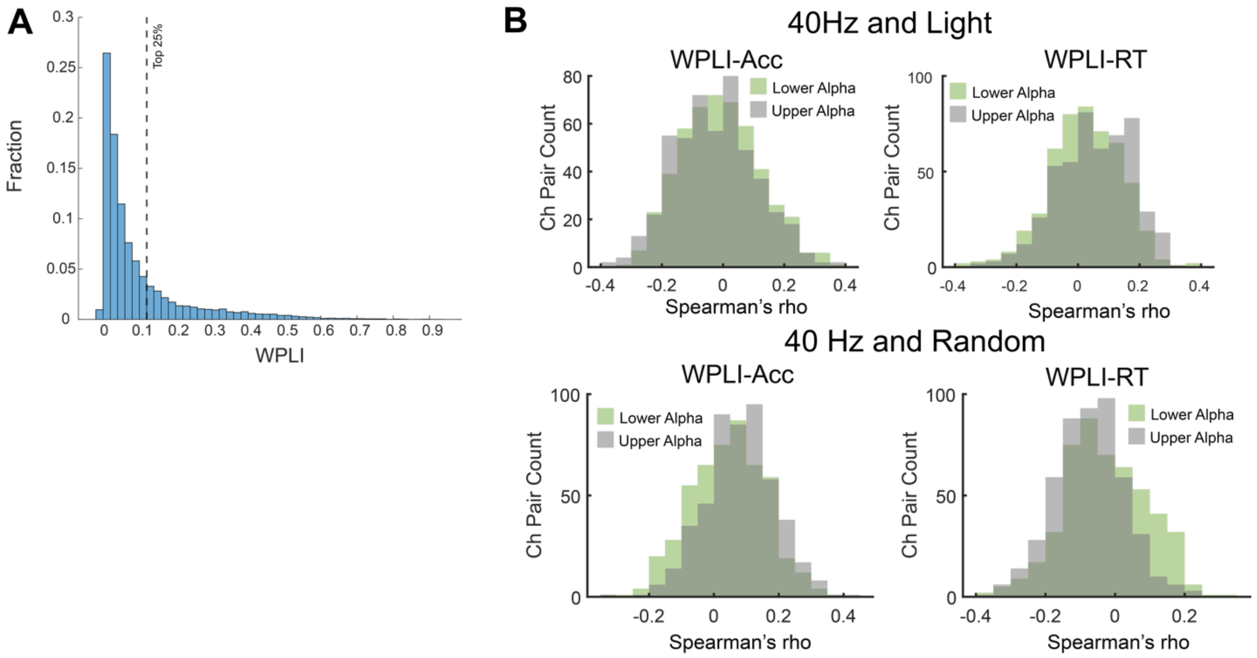

Supplementary Figure 4. WPLI and WPLI-behavior correlation distributions

(A) Distribution of peak WPLI values within the alpha band of all subjects' channel pairs with top quartile (WPLI = 0.12) indicated by dashed line (n = 67 subjects x 496 channel pairs = 33,232 alpha peak WPLI values).

(B) Distribution of WPLI-Behavior Spearman's rank correlation coefficients (rho) across all channel pairs (n=496) comparing lower alpha (green) and upper alpha (grey) frequency bands. *Top left*: Distribution of WPLI-Accuracy Spearman's rho values for the 40 Hz and Light conditions. The distributions of lower alpha and upper alpha were significantly different (paired t-test, $p = 0.003$). *Top right*: Distribution of WPLI-Reaction Time Spearman's rho values for the 40 Hz and Light conditions. The lower alpha and upper alpha distributions were significantly different (paired t-test, $p = 4 \times 10^{-9}$, n=496). *Bottom left*: As in top left for 40 Hz and Random conditions. The lower alpha and upper alpha distributions were significantly different (paired t-test, $p = 5 \times 10^{-10}$, n=496). *Bottom right*: As in top right for 40 Hz and Random conditions. The lower alpha and upper alpha distributions were significantly different (paired t-test, $p < 10^{-16}$, n=496).
